# Supplementary figures and images for: Optimal sequence-based design for multi-antigen HIV-1 vaccines using minimally distant antigens
Source: PLoS Comput Biol. 2022 Oct 31;18(10):e1010624. doi: 10.1371/journal.pcbi.1010624 (PMC9621458; doi:10.1371/journal.pcbi.1010624)

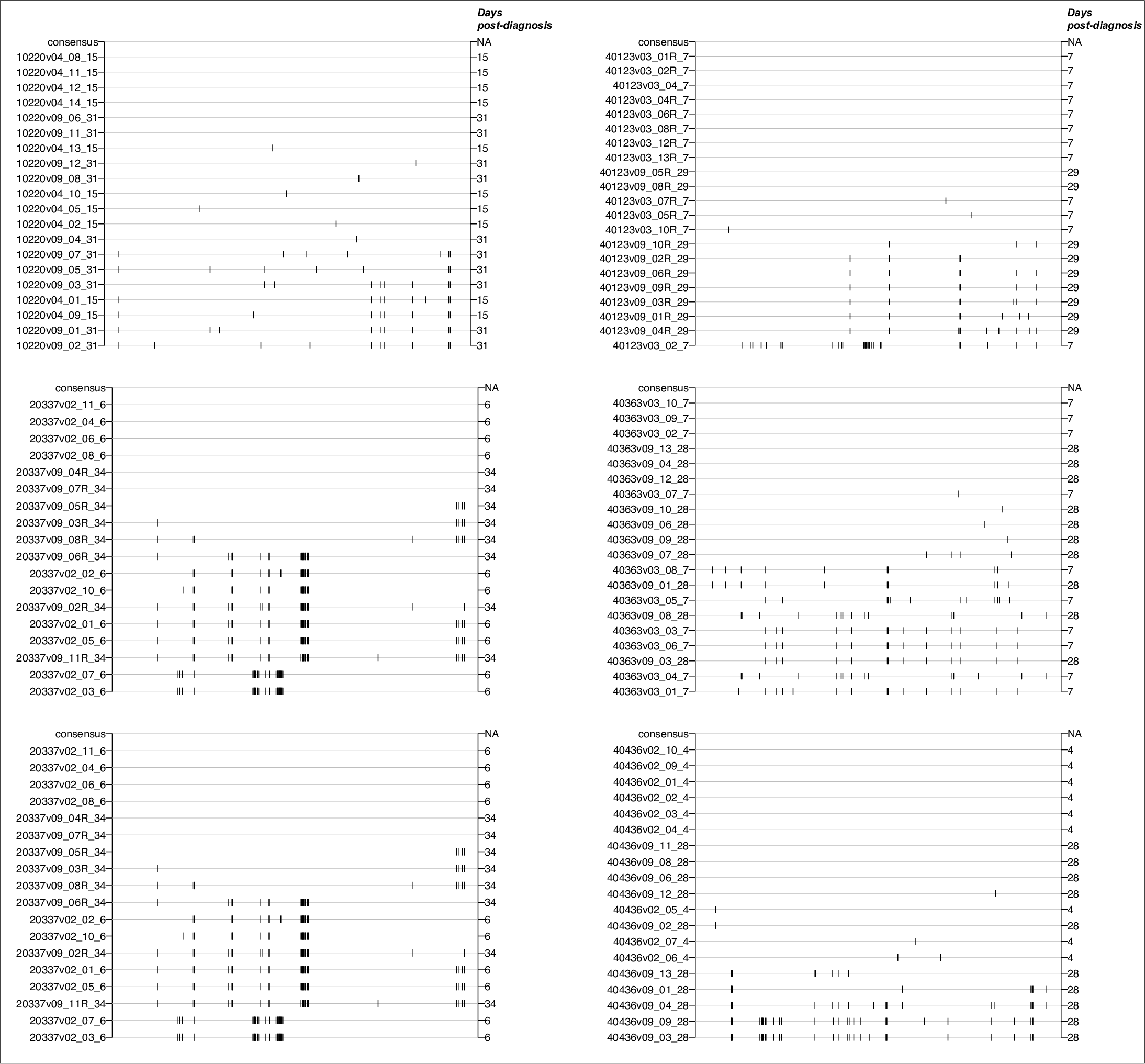

Supplement: S1 Fig — For each individual, a highlighter plot is constructed from sequences sampled during acute infection using the consensus as the master sequence. The number of days post-diagnosis at which each sequence was sampled is listed to the right of each plot. (TIF) [file pcbi.1010624.s001.tif]

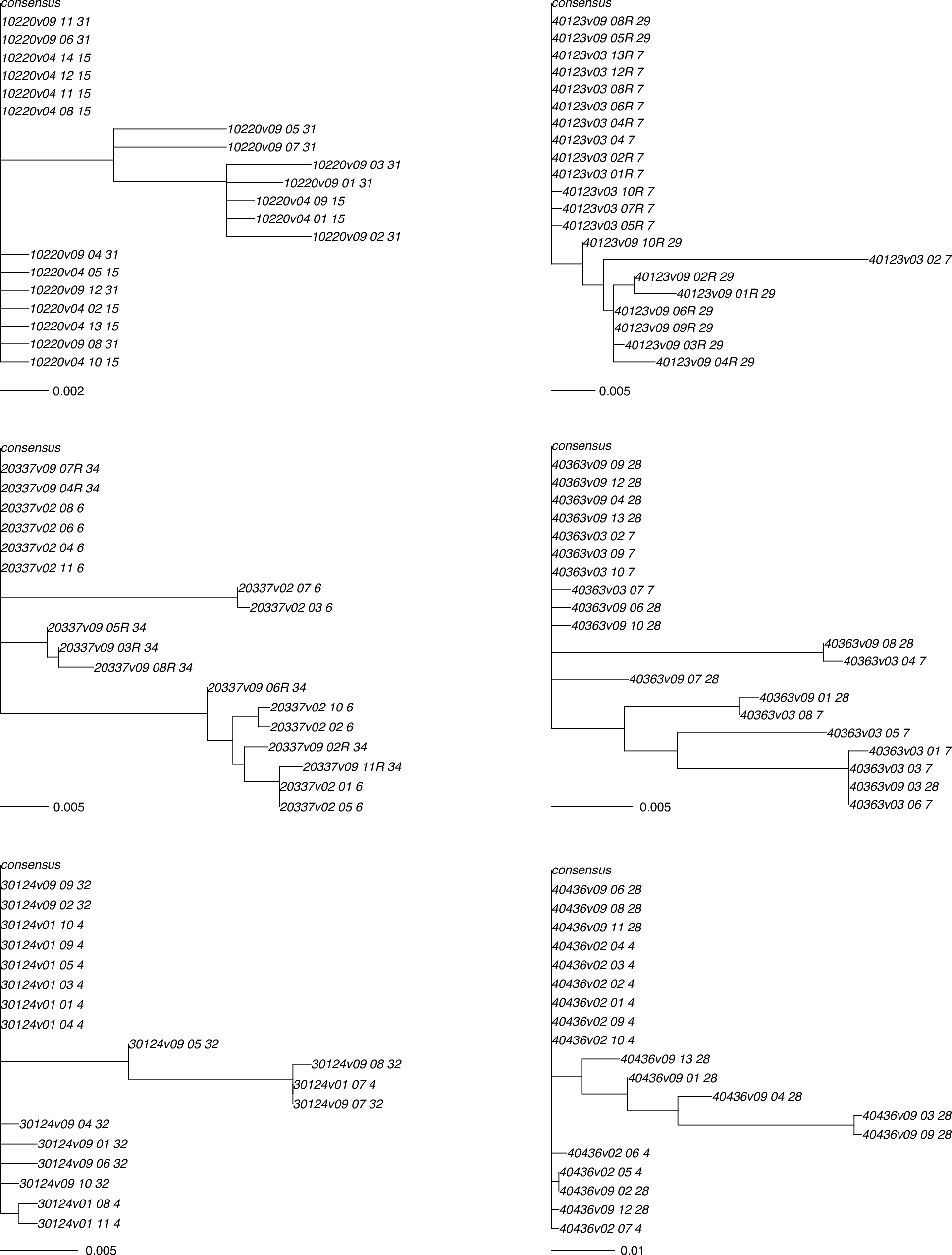

Supplement: S2 Fig — For each individual, a phylogeny constructed from sequences sampled during acute infection and rooted on the majority consensus sequence. (TIF) [file pcbi.1010624.s002.tif]

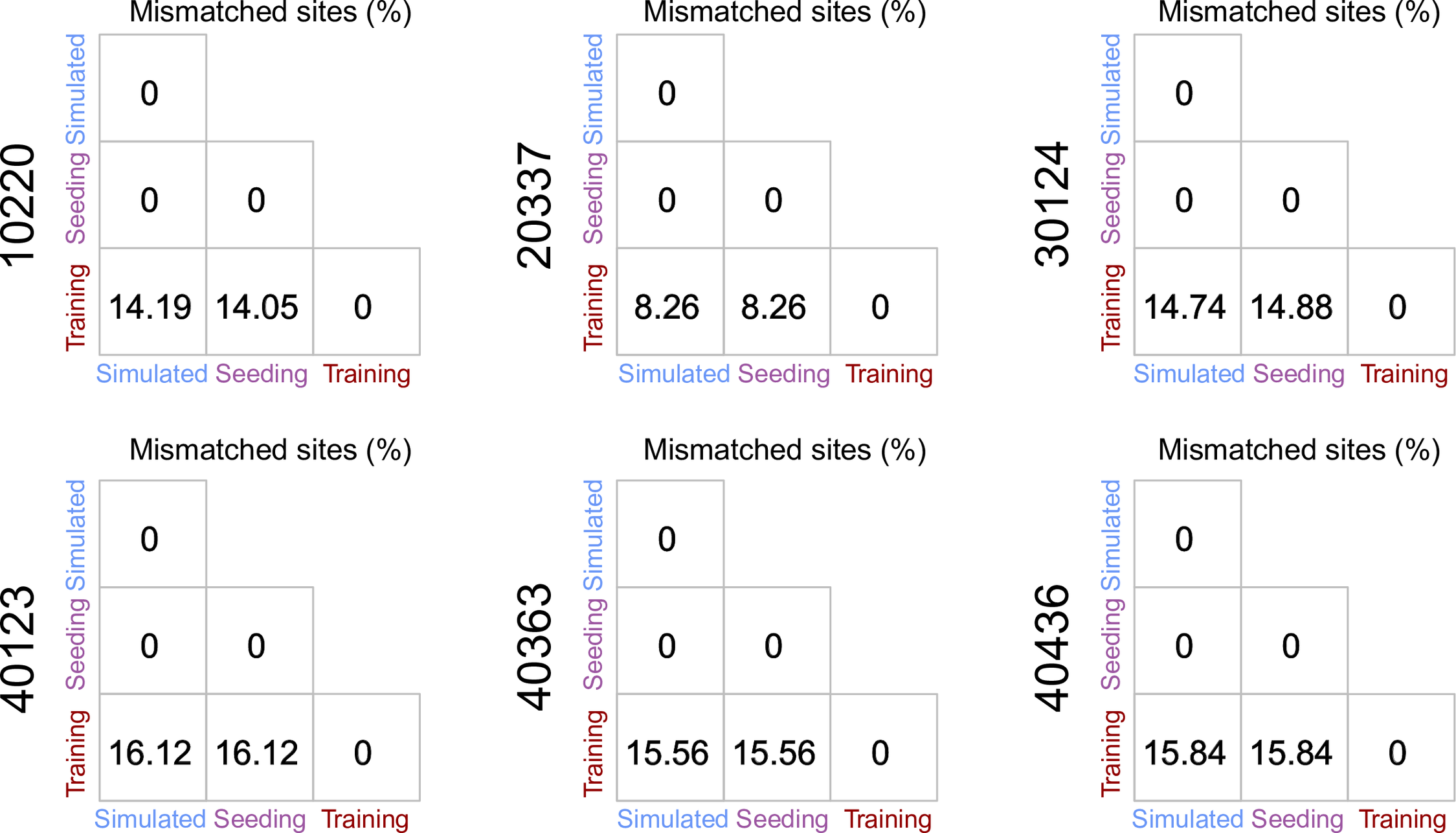

Supplement: S3 Fig — Correlation plot of the percentage of mismatched non-gapped sites between the consensus of the seeding alignment, simulated alignment, and training alignment for sequences simulated under each RV217 multi-founder infection. (TIF) [file pcbi.1010624.s003.tif]
