# Supplementary material for: Optimal sequence-based design for multi-antigen HIV-1 vaccines using minimally distant antigens
Source: PLoS Comput Biol. 2022 Oct 31;18(10):e1010624. doi: 10.1371/journal.pcbi.1010624 (PMC9621458; doi:10.1371/journal.pcbi.1010624)
Supplement: Attachment — Submitted filename: Response_to_reviewers_20220815.docx [file pcbi.1010624.s005.docx]

**Reviewer #1:** Lewitus et al describe a method to simulate HIV antigenic diversity using a hidden Markov model, intended to replicate the diversity found in individuals who produce broadly neutralising antibodies. The intended application is future vaccine design. This is naturally a potentially worthwhile direction of research and a potentially fruitful approach. Unfortunately the presented manuscript has simply too many unanswered questions for me to be able to recommend publication.

We appreciate the reviewer’s perspective that this is a worthwhile approach to designing HIV-1 vaccine immunogens. There were indeed multiple aspects of the original manuscript that were unclear that we have made efforts to address in the significantly revised manuscript.

Unanswered question #1: What is the actual model here? As a reader unfamiliar with how HMMs are used in this field, the description of how the model works (L458-482) is unsatisfactory. Neither the underlying process nor the observation process are described. Step 1 is simply “a transition rate matrix was estimated”, but how was it estimated, and indeed, what are we transitioning between? Is this a model where evolution happens over time, or between subsequent sites on the genome? Nowhere in the paper is this made clear. The mathematical notation is equally confusing, e.g. “n_k is the number of occurrences” – the number of occurrences of what? Why does theta’, which is (I think) a matrix, occur multiple times as a constant in the multinomial p.m.f.? With j defined as a site and k as an amino acid, how can we ever have j=k? This is entirely incoherent. It makes it impossible for me to properly evaluate the potential strengths and weaknesses of the model. I accept that this may be one of a standard class of HMMs which I am not familiar with – but no other example is cited, and even if this is true it does not excuse the failings of the notation in this manuscript.

We thank the reviewer for pointing out these issues. We rewrote the model description and no longer refer to it as an HMM. We made significant revisions in different parts of the manuscript:

In the Results section:

“The model was based on two training alignments, $F_{1}$ and $F_{2}$. $F_{1}$ corresponded to all the sequences descended from the major founder variant, i.e. the major founder lineage in a participant infected with multiple founder variants. $F_{2}$ grouped all sequences descended from minor founder variants in that participant (**Fig 1A**). Infections with multiple founder variants can be established with two founders that are clearly delineated; however, rare lineages and recombinant forms (based on the extant or unsampled founder variants) are often identified - these are sometimes found as singletons. While the model can be adapted to n founder lineages, for simplicity, here we considered the major founder lineage $F_{1}$ and grouped the other sequences in that participant (all closely phylogenetically related) as representing $F_{2}$. Hence, a group is not necessarily comprised of sequences descending from a unique founder variant but represents a genetically differentiable cluster with a divergent evolutionary pattern; identifying and distinguishing such clusters allows to recapitulate the diversity of acute infections with multiple founders without biasing towards one founder variant population. The percentage of non-consensus residues at each site ($\pi_{j}$) was calculated for $F_{1}$ and $F_{2}$ separately, giving a mutational landscape for each founder lineage (major and minor viral population) (**Fig 1B**). Next, a transition matrix, $\Theta$, was required to define the probability of each amino acid (or gap) transitioning into any other amino acid (or gap) (**Fig 1C**); the transition matrix is derived from a large set of independent circulating HIV-1 sequences (sampled from LANL HIV database). Finally, a single sequence, aligned to the training alignment reference frame, was required to seed the model (**Fig 1D**). The single seeding sequence corresponds to the design target and can be any sequence for which a set of multi-founder like antigens is to be derived, e.g. the consensus (or most common recent ancestor) from a set of sequences corresponding to a particular participant or to a specific HIV-1 subtype or Circulating Recombinant Form (CRF). At each iteration, $n$, site $j$ of the seeding sequence had a probability of mutating proportional to $\pi_{j}$ to a new residue defined by $\Theta_{j}$, where $\pi$ was randomly drawn from either $F_{1}$ or $F_{2}$ (**Fig 1E**). The model then outputs simulated sequences in an alignment, wherein equal numbers of sequences are derived from each founder lineage model (**Fig 1F**). Instead of considering the variability in a given participant, the model can also rely on a pooled dataset of major vs. minor founder lineages from multiple participants. In that case, at each site, the mutational pattern can be that of any individual in the pooled set (to retain within-host diversity characteristics and forbid inter-host transitions).”

In the Methods Section:

“A probabilistic model was developed to simulate Env sequences that replicated the variability of infections with multiple founder variants. The model is trained on two Env alignments, corresponding to the major (*F1*) and minor (*F2*) founder lineages identified in a given participant (the model can also be trained on a pooled set of individual lineages). A transition probability matrix, $\Theta$, is estimated by directly computing the transition rate between pairs of amino acids, including transition rates from/to gaps based on an alignment of empirical sequences. We suggest using a large dataset of independent sequences that would correspond to the subtype of the desired target vaccine candidate (i.e., subtype C sequences if the goal is a subtype C vaccine which implies that the seed sequence corresponds to subtype C). For each alignment,

1. The percentage of non-consensus residues was calculated for each alignment site.
2. For each site, $j$, percentage of non-consensus residues, $\pi_{j}$, an amino acid in the seeding sequence, $k$, and a transition probability, $\Theta_{k}$, the probability of transitioning from $k$ to $k’$ was written as

$$P\left( k'|k,\pi_{j},\Theta_{k} \right)=\frac{\Theta_{k}\left[ k \right]\pi_{j}}{\Sigma\left( \Theta_{k}\left[ -k \right] \right)}$$

where $\pi_{j}$ has an equal probability of being defined by $F_{1}$ and $F_{2}$. The new residue is then drawn from a multinomial probability distribution based on $P_{n}$. For each seeding sequence (e.g., a subtype C consensus sequence), whichever model is initially drawn to simulate the initial amino acid in a sequence is consistently applied to generate the following amino acids in that sequence. For the pooled sets, the less diverse major founder lineages were pooled together and the more diverse minor founder lineages constituted a second set. For each pooled founder lineage alignment, at each site j, $\pi_{j}$ was estimated as the maximum percentage of non-consensus residues in any individual alignment in that pool (i.e., this retained within-host diversity levels); however, this could be alternatively modeled such that, at each site j, $\pi_{j}$ was randomly drawn from an individual alignment in the pooled alignment.”

Unanswered question #2: What is to be made of the difference in variances in divergence and pairwise distance between the simulated and actual (multiple) sequences in figure 4? I presume that the reported p-values are for t-tests (i.e. equality of means); it seems highly unlikely that p>0.05 would be obtained if testing for equality of variances. It would seem that there is potentially much more diversity in real data than this model can readily capture in simulations. In that case, how generally useful is this?

We recognize that the comparisons here were limited and have expanded our analyses in the revised manuscript. Indeed, the variance simulated by our approach when it is trained on one participant (e.g., 40363) will not generate the variance observed across multiple participants, as was shown in Figure 4 (now Fig 3). In the revision, the model is trained on six participants (with infections with multiple founder variants) separately and, as a new feature, the training model combined these six participants to create a pooled dataset (as mentioned in the model description above). The Results section and Figures 2 and 3 describe these results:

“An empirical transition probability matrix, $\Theta$, was computed on an alignment of 172 subtype C Env sequences sampled since 2011 and simulations were seeded with the consensus derived from sequences from an independent RV217 participant (id=10066). One thousand sequences were simulated trained on major and minor founder lineages alignments for each participant separately. The percentage of non-consensus residues in simulated sequences at each site was highly correlated (median $R^{2}$=0.99, min=0.98, max=100) with the percentage in the training alignment for each founder lineage (**Fig 2C,D**). One thousand sequences were also simulated while trained on a pool of all founder lineage alignments across participants (**Fig 2E**), where $F_{1}\left( \pi_{j} \right)$ was the maximum percentage of non-consensus residues found at site j in a given individual across all $F_{1}$ alignments and $F_{2}\left( \pi_{j} \right)$ was the maximum percentage of non-consensus residues found at site $j$ in a given individual across all $F_{2}$ alignments. The 95% confidence intervals of median pairwise diversity in simulated sequences included median pairwise diversity of training alignments for sequences simulated on each participant and for the pooled set of participants (**Fig 2F**). Similarly, the 95% confidence intervals of the mean number of polymorphic sites per simulated sequence as well as the number of polymorphisms per sequence at CD4bs, V1-V2, V3 and MPER contact sites included the mean number for training alignments for sequences simulated on each participant (**Fig 2G**).”

“We compared diversity and divergence estimates for sequences simulated under the pooled set of major and minor founder lineage alignments to Env sequences sampled during acute infection from RV217 participants infected with either a single founder variant (and who developed <35% neutralization breadth) (n=12) or multiple founder variants (and who developed >70% neutralization breadth) (n=6). The 95% confidence intervals of diversity and divergence estimates for simulated sequences included the median values for Env alignments of multi-founder variant acute infections and was higher than that of infections with single founders (**Fig 3A-D**).”

The comparison tests used were Mann-Whitney U tests, specifically because we wanted to avoid testing for similarities in means. However, per the reviewer’s comment below we have removed these comparison tests for simulated data in the revised manuscript and only reported median values and interquartile ranges, as comparison tests are not generally appropriate for simulated data (as the reviewer states below). The Methods section was revised as follows:

“Shapiro’s normality test was used to determine if data were normally distributed. If data were normally distributed, pairwise comparisons were made using a Student’s t-test; and otherwise using a Mann-Whitney U test. Two-sample Kolmogorov-Smirnov tests were used to compare distributions. Statistical tests were only used to compare empirical data but not simulated data. Comparisons with simulated data were made by assessing inclusion/exclusion of values within the 95% confidence intervals of simulated data.”

Unanswered question #3: Is the phylogenetic pattern of one very diverse collection of sequences and one not very diverse collection typical of those samples identified as multiple founder? I would not say, on the face of it, that figures 5A or 6D look like multi-founder infections; in fact it rather looks like viruses in “founder 2” are closer to those in “founder 1” than they are to each other. So what actually links those in “founder 2” to each other? Why does that pattern not suggest that all samples are descendants of a single founder strain with a bimodal distribution of subsequent mutations? Are the authors sure that they have robustly identified infections with multiple founder strains, as the one presented example seems to beg a lot of questions? If the multiple-founder status of individual 40363 is established in another way, the question remains – is this pattern typical?

We agree that the previous description was insufficient and problematic. We provide in the revised manuscript further description of previous literature on multiple founders and an expanded description of the multiple founders in the six infections we considered. While we can confirm that the infection had multiple founder variants, enumerating the number of distinct founders is difficult. Therefore, we revised our approach to describe a major and a minor founder lineage. We grouped distinct sequences together as the minor founder lineage to simplify our description. In addition, because we are not considering the founder variant per se but all the sequences descended from it after a few weeks of viral replication (typically about one to two weeks under the eclipse phase plus two weeks post diagnosis), we refer to both populations as founder lineages. In addition, the trees and summary description of the alignments are shown for each participant used iin the training model as supplementary figures. The text was revised in multiple sections of the manuscript.

In the Introduction:

“Infections with multiple HIV-1 founder variants account for approximately 25% of infections [25, 34-37]. These multi-founder infections occur when multiple sequences are transmitted from a single transmitter who was likely in the chronic phase of their infection. Hence, all the sequences in the recipient are closely related, phylogenetically linked and show around 1% intra-participant diversity.”

In the Results:

“Infections with multiple founder variants can be established with two founders that are clearly delineated; however, rare lineages and recombinant forms (based on the extant or unsampled founder variants) are often identified - these are sometimes found as singletons.”

As a final comment: the use of p-values in a simulation study is always a little unsatisfactory because if a difference actually exists, any desired p threshold can be met by simply doing more simulations.

We agree that removing comparison tests and reporting on median and IQRs is better practice for simulated data, which we have done in the revised manuscript. To compare simulated data to empirical data, we report on whether median values of empirical data fall within the 95% confidence intervals of simulated data (see above response).

**Reviewer #2:** Lewitus et al describe a method for 'evolving' HIV env populations in silico from a seed sequence, where the goal is to simulate the type of within-host diversity that naturally occurs after infection with more than one founder variant. The authors propose that selected sequences from these in silico-evolved populations could be used as (multi-valent) HIV vaccine antigens, to elicit superior antibody neutralization breadth. This vaccine antigen design approach is supported by recent work by the authors, currently under review, showing that individuals with multiple founder variants were more likely to develop neutralization breadth years later. The authors conclude their results section by applying their method to "evolve" a subtype C consensus sequence, and propose that the approach could be applied to any subtype (or recombinant) consensus to generate subtype-specific vaccine antigens.

This is a novel approach that addresses an important health problem, but I have a number of concerns, which, if founded, could considerably reduce the relevance of the approach.

We thank the reviewer for their constructive comments and want to add that the manuscript that forms the basis of this work is now published:

<https://journals.plos.org/plospathogens/article?id=10.1371/journal.ppat.1010369>

1. Dual-founder infections are initiated by two different (yet still closely related) sequences (as both founders are transmitted from the same donor). But, the authors do not seed their simulations with two different founders. Rather they start with a single seed, and 'evolve' it under two different rates, to produce descendant populations.

We chose a single seed sequence based on the rationale that in infections with multiple founder variants, the most recent common ancestor of all the sequences sampled in the acute infection is typically relatively recent. It is also important to start with a single sequence because we want to create a generalizable approach for a standard vaccine. Previous data show that a centralized sequence, such as a consensus or MRCA, are more representative of HIV-1 circulating viruses than any independent sequence. Hence, our approach is suitable to make a subtype specific vaccine where we would start with a consensus sequence for that subtype (as we suggest with subtype B). While we could envision taking the consensus of each variant (and evolving each individually) if we were considering sequences from a given participant with a multi-founder infection as the basis for a vaccine candidate, this approach would not allow us to start directly from an optimal subtype specific consensus.

The text was modified as follows:

“The single seeding sequence corresponds to the design target, it can be any sequence for which a set of multi-founder like antigens is to be derived, i.e. the consensus (or most common recent ancestor) from a set of sequences corresponding to a particular participant or to a specific HIV-1 subtype or CRF.”

These rates are based on empirical data from an individual with a dual founder infection (40363), and the approach does yield diverse descendant populations. But, the second rate, which was computed based on the diversity in the sequences that were identified as the descendants of 40363's second founder virus, seems unbelievably high. Is 40363's dataset a really "representative" one to use for this purpose? Perhaps more importantly, is this extremely high rate even accurate? Looking at the partial phylogeny of 40363's sequences (Figure 5A, bottom) I wondered if the sequences that were identified as the descendants of their second founder virus might in fact comprise two populations, namely: the *actual* descendants of the second founder virus (three sequences at the bottom of this tree) and recombinants of the first and second founders (the three divergent sequences in the middle of this tree). This indeed would produce a transition rate matrix with very high the rate. But, evolving sequences this way may not actually yield appropriate vaccine antigens.

The reviewer raises two important points that we sought to address in the revised manuscript.

First, while our now published work on single- and multi-founder infections in the RV217 cohort (Lewitus et al., PLoS Pathogens, 2022) shows that the diversity in 40363 during acute infection is representative of a median diversity compared to other infections established with multiple founder variants, we agree that using only 40363 to train the model was problematic. In the revised manuscript we train the model on infections with multiple founders from six participants separately and also train the model using a pooled dataset that includes the six participants. The revised Figure 2 shows results for individual participants and for the pooled set.

**Fig 2: Simulated sequences reproduced the variability of acute HIV-1 infections with multiple founder variants.** (A) A highlighter plot and (B) phylogeny for Env sequences sampled at 7 and 28 days post-diagnosis from a RV217 participant (id=40363) with an infection with multiple founder variants. (C) The percentage of non-consensus residues at each Env site in sequences simulated from the major founder variant or major lineage 1 (top, gold) and the minor founder variants grouped here as lineage 2 (bottom, blue) after seeding with the consensus sequence from an independent acutely-infected RV217 participant (id=10066); values for sequences belonging to the major and minor founder lineages in 40363 are shown in red. (D) Regression plots of the percentage of non-consensus residues in the training alignment as a function of non-consensus residues in the simulated alignment for (top, gold) founder lineage 1 and (bottom, blue) founder lineage 2. (E) Phylogeny of sequences sampled at 4-34 days post-diagnosis from 6 RV217 participants with infections with multiple founder variants. Tips are colored to represent the population corresponding to the major and minor founder populations for each participant (for simplicity, multiple founder variants or singleton sequences are grouped under the minor lineage). (F) For sequences simulated under each training alignment (see panel E), the pairwise diversity of the training alignment (red) and of the sequences simulated under that training alignment (blue); and the pairwise diversity of sequences simulated under the pooled alignment (blue) are shown. Solid lines represent 25% and 75% interquartile ranges. (G) Barplots of the number of polymorphic sites per sequence in sequences simulated under each training alignment and the pooled-participants training alignment (blue) at all sites, CD4bs, V1-V2 contact sites, V3 contact sites, and MPER sites. Dashed lines indicate maximum values. Red dots represent median values for training alignments.

Second, we agree that using the alignments from each 40363 founder variant to calculate the transition rate matrices was not appropriate. In the revised manuscript, we compute a single transition rate matrix defined by a large alignment of representative subtype C sequences and advocate for using a population-level alignment matching the vaccine candidate target. The text was modified as follows:

“A transition probability matrix, $\Theta$, is estimated by directly computing the transition rate between pairs of amino acids, including transition rates from/to gaps based on an alignment of empirical sequences. We suggest using a large dataset of independent sequences that would correspond to the subtype of the desired target vaccine candidate (i.e., subtype C sequences if the goal is a subtype C vaccine which implies that the seed sequence corresponds to subtype C).”

This is worth addressing because the authors identify a "bimodal phylogenetic topology" as a key correlate of eliciting bNabs (line 376). Dual founder viruses however will produce a specific type of bimodal tree topology because the two founder sequences are different (as mentioned above there may also be recombination between descendant founders, yielding populations that look "intermediate" in a tree). But the bimodal phylogenies produced by the authors' approach could be of a very different type than 'natural' ones. The partial trees shown in 5A, and the presentation of results as tree summary statistics (where topological information is lost) is insufficient to alleviate my concern. (note: the authors do train the model on founder variants from six other participants, though it is not specified whether these also had widely different rate matrices like 40363's did, and they do present favorable summary statistics, but again no trees are shown).

We removed the notion of bimodal topologies as associated with neutralization breadth since this is not a specific condition we tested in our published work. While many infections with multiple founders consist of two variants and hence have bimodal phylogenies, there are often recombinant sequences or rare lineages that break this simplification as the reviewer noted.

We provide phylogenies for the six participants with multi-founder infections in **S2 Fig** in the revised manuscript. Nonetheless, the summary statistics that were presented in Figure 5A and now correspond to **Fig 3F-H** retain topological information. We used three tree summary statistics that can be used to describe tree topologies, as we previously showed in Lewitus & Morlon, Syst Bio, 2016 and Lewitus & Rolland, Virus Evolution, 2020:

“Similarly, phylogenies of simulated sequences were more similar to phylogenies of infections with multiple founder variants than to those with single founder variants (**Fig 3D**). Spectral density profiles of the modified graph Laplacian [39] were computed for Env alignments from infections with single and multiple founder variants in RV217 and for down-sampled alignments from sequences simulated under the pooled set of major and minor founder lineages alignment. Spectral density profile summary statistics each capture a unique aspect of phylogenetic topology: l* is proportional to non-synonymous/synonymous rates, h is inversely proportional to transversion-transition rates, and yis proportional to rate heterogeneity [40]. The simulated alignment was downsampled 100 times for 5 sequences simulated under each of the founder lineage training alignments, F_1_ and F_2_. The 95% confidence intervals of l*, h, and ln-transformed y for simulated sequences included the median values for Env alignments of acute infections with multiple founder variants but none included the median value for infections with a single founder variant (**Fig 3F-H**). “

To summarize, the authors should address:

- why they chose to simulate multiple founder descendant populations by applying different mutation rates to a single founder (rather than applying similar rates to distinct yet closely related founders)

As we stated above, we chose a single sequence because we want to create a generalizable approach that will allow us to design a subtype C vaccine candidate, and the goal of our approach is to create multi-founder like ‘noise’ around a central sequence that we deem an ideal vaccine target. We consider that this can also be justified by the fact that the most recent common ancestor in a given infection with multiple founder variants is typically relatively recent, i.e. corresponding to a time span that is usually shorter than the duration of infection in the previous host.

- whether the vastly different rates for 40363's two founder populations are truly representative

In the revised manuscript we no longer calculate the transition probability matrix from 40363 sequences (and use a population level set instead) and also include a pooled set comprising the six alignments from RV217 participants with multi-founder infections to train the model:

- whether the phylogenies inferred from the simulated sequences are topologically similar to sequences sampled from real dual-variant infections.

We sought to more carefully describe in the text how using summary statistics from the modified graph Laplacian captures topology and is therefore appropriate for comparing phylogenetic structures as mentioned above. This is also described in the Methods section:

“The modified graph Laplacian (MGL) is computed for the distance matrix of the reconstructed phylogeny of sequences; eigenvalues calculated from the MGL define the connectivity of the phylogeny in terms of substitutions. Spectral density profile summary statistics represent different aspects of the topology of the phylogeny, such as the longest path through the phylogeny, $\lambda*$ which is a correlate of non-synonymous/synonymous substitution rates, the proportion of long versus short branching-events, $\psi$, which is a correlate of rate heterogeneity, and the occurrence of branching-events, $\eta$, which is a correlate of transition-transversion rates [39, 40]. Spectral density profile summary statistics $\lambda*$, $\psi$, and $\eta$ were estimated for phylogenies reconstructed from empirical and simulated sequences.”

- the potential implications of recombination in the empirical data, as well as the approach
We agree with the reviewer that this is an important concern. Recombination is so pervasive in HIV-1 that there are recombinant sequences in our dataset and these recombinant patterns would be captured in our approach. Our sequence set lacked the sequencing depth to characterize this precisely. However, as the reviewers pointed out, some sequences appeared to be potential recombinants. We modified the text to describe that infections with multiple founders often included recombinant sequences:

“Infections with multiple founder variants can be established with two founders that are clearly delineated; however, rare lineages and recombinant forms (based on the extant or unsampled founder variants) are often identified - these are sometimes found as singletons.”

2. Though the authors indicate that individuals with dual-founder infections are more likely to eventually produce bNAbs, this takes years because these antibodies are produced as a result of ongoing co-evolutionary process where Abs adapt to ever-evolving immune-escape variants in vivo (and some current clinical trials are sequentially immunizing with increasingly "evolved" immunogens to recapitulate this). The authors' approach however appears to design immunogens based on the immediate descendants of the founder viruses only, but does not address the issue of time, nor this biological process of Ab/HIV co-evolution. Or, are the authors proposing that this approach could be used to "evolve" immunogens in silico to generate sequential immunogens? This question ties into my question 3d) below regarding whether this process is really a Markov chain.
We agree that at this stage we are mostly concerned with the priming vaccination. This stems from our findings that the initial events in infection in RV217 participants were key for the development of breadth, those with infections with multiple founders and/or with Env that showed superior engagement of B cells in the months developed neutralization breadth.

We seek to reproduce that initial favorable step with our vaccine design. We do not have a set plan for an optimal boosting strategy and want this choice to be guided by experimental data. We will initiate experimental assays in animal models to test our designed sequences as a cocktail of five immunogens that is used in a prime/boost series with three sequential immunizations. We are also designing experimental assays where the boost will be different from the priming cocktail.

We revised the discussion to include a description of where our design fits when compared to more advanced HIV-1 vaccine strategies:

“In silico vaccine design methods have been used for HIV-1 for over two decades to overcome the obstacle posed by HIV-1’s extreme diversity [60-62]. There is more diversity within each HIV-1 subtype or CRF than what can be seen across a viral species [63]. Gaschen and colleagues showed that a centralized sequence, consensus or ancestral, would better represent HIV-1 than any sequence derived from an HIV-1 infected person [61, 64]. To cope with HIV-1 diversity, some designs, such as the mosaic approach, seek to integrate a fraction of the diversity seen in HIV-1 sequences [65-67]. These variability-inclusive strategies are reminiscent of the diversity seen in super-infections, which have previously been associated with the development of neutralization breath. As such, mosaic antigens are designed to be maximally distant to cover a large fraction of circulating viruses. The rationale is that immunizations with these diverse mosaic inserts, for example corresponding to consensus sequences for group M, subtype B and subtype C, could lead to the development of antibody responses against these distant viruses thereby potentiating broadly cross-reactive responses. Two vaccine efficacy trials are testing the Mosaic design (one reached futility criteria in 2021: https://www.jnj.com/johnson-johnson-and-global-partners-announce-results-from-phase-2b-imbokodo-hiv-vaccine-clinical-trial-in-young-women-in-sub-saharan-africa). An opposite strategy to Mosaic designs was to focus on only the most conserved elements of HIV-1 [68-71]. This stemmed from the realization that variable segments of HIV-1 functioned as decoys eliciting immune responses that were not optimal and that only a small fraction of HIV-1 diversity could be integrated in a vaccine candidate of practical size. Another currently leading strategy, the germline targeting approach, seeks to improve the longitudinal process seen in individuals infected who later developed breadth [72] by reproducing the directional process that leads to breadth in a minority of individuals through using antigens that correspond to stepwise stages of the co-evolution between the virus and the neutralizing response. Our approach is also based on a process seen in natural infections, whereby infections with multiple founder variants were linked to the subsequent development of neutralization breadth. This multi-founder-like design of minimally distant antigens is also akin to the conserved elements vaccine design rationale. We consider that the ‘noisification’ of a central consensus sequence at target sites for key antibodies will trigger responses to these antibody epitopes and that the toggle between these minimally distant epitopes will promote a desirable affinity maturation process leading to the development of bnAbs.”

3. The paper would benefit from additional explanation of key biological and computational concepts to make it more accessible to readers with diverse expertise. Specifically:

a) Can the authors define what they mean by "minimally distant antigens"? I suspect that lines 408-410 contain the necessary information, but these lines don't come until the paper's conclusion. Adding to this confusion is that the authors, in their evolution of the subtype C strain, specifically select four *maximally diverse* sequences, presumably as their candidate vaccine antigens.

We thank the reviewer for this suggestion. We clarified what we meant by minimally distant antigens across the different sections but especially in the Introduction. We hope that the improved description of what infections with multiple founders are in the revised manuscript should also help readers.

Line 31 (Summary):

“By training an HIV-1 Env consensus sequence on the diversity from acute infections with multiple founders, we derived in silico a set of minimally distant antigens that is representative of the diversity seen in a multi-founder acute infection.”

Line 79 (Introduction):

“This led us to propose that a vaccine which would be constituted by multi-founder like, minimally distant antigens (differing by ~1% in Env) could initiate the induction of bnAbs.”

Line 87 (Introduction):

“Here we present a probabilistic model for simulating Env alignments that resemble a set of minimally distant antigens representative of the variability seen in multi-founder acute infections.”

We also clarified that the *maximally diverse* selection criteria was applied within the minimally distant space, i.e. to best cover the minimal genetic space corresponding to infections with multiple founder variants. The text was modified as follows:

Line 282 (Results):

“From these, two candidate sequences generated by $F_{1}$ and two by $F_{2}$ that were maximally divergent (within the framework of minimal diversity) were selected as candidate antigens (**Fig 5G**, **File S1**).”

Line 1903 (Methods):

“To select candidate antigen sequences, simulated sequences were filtered by those that had a non-consensus residue (with respect to the subtype C consensus) in at least three key Ab epitopes (VRC01, CAP256-VRC26.25, PGT121, 10E8, and 35O22). Of these sequences with minimal variability, the two maximally divergent sequences simulated by $F_{1}$ and two by $F_{2}$ were selected as candidate antigens. Maximally divergent sequences were selected to cover as much genetic space as possible within the simulated minimal divergence.”

b) The authors should explicitly state, early on, that multi-founder virus infections happen when more than one donor sequence is transmitted to the recipient (and therefore that these viruses, though distinct from one another, are still quite closely related). While this will be obvious to experts, failing to state this could confuse non-experts, particularly as the authors mention superinfection and within- and between- subtype HIV diversity (where the scale of this type of diversity are far, far higher than that seen in a dual- or multi-variant infection).

We corrected the introduction to describe these infections and provided additional details in the Results section. We also hope that the new discussion helps put our minimal variance approach in perspective when compared to current vaccine strategies.

The introduction was modified as follows:

“Infections with multiple HIV-1 founder variants account for approximately 25% of infections [25, 34-37]. These multi-founder infections occur when multiple sequences are transmitted from a single transmitter who was likely in the chronic phase of their infection. Hence, all the sequences in the recipient are closely related, phylogenetically linked and show around 1% intra-participant diversity.”

c) The authors should avoid using the term "founder variant" to refer to BOTH the founder variant and its descendants. Use a different term for the latter. Also, the authors should state explicitly how the founder virus sequence is inferred from its descendant populations (by taking the population consensus of early descendants)

We agree with the reviewer that this was problematic. We also agree that the founder variant and its descendants are distinct things. Here we are concerned with the lineage defined by the descendants from each founder. The founder variant itself would be approximated with a consensus sequence of all the sampled sequences corresponding to that variant. Since we do not use in our design these derived consensus sequences for each founder, we do not specify them in the manuscript. The text was revised as follows:

“The model was based on two training alignments, $F_{1}$ and $F_{2}$. $F_{1}$ corresponded to all the sequences descended from the major founder variant, i.e. the major founder lineage in a participant infected with multiple founder variants. $F_{2}$ grouped all sequences descended from minor founder variants in that participant (**Fig 1A**). Infections with multiple founder variants can be established with two founders that are clearly delineated; however, rare lineages and recombinant forms (based on the extant or unsampled founder variants) are often identified - these are sometimes found as singletons. While the model can be adapted to n founder lineages, for simplicity, here we considered the major founder lineage $F_{1}$ and grouped the other sequences in that participant (all closely phylogenetically related) as representing $F_{2}$. Hence, a group is not necessarily comprised of sequences descending from a unique founder variant but represents a genetically differentiable cluster with a divergent evolutionary pattern; identifying and distinguishing such clusters allows to recapitulate the diversity of acute infections with multiple founders without biasing towards one founder variant population.”

d) It is not clear that the use of "Markov chain" and "hidden Markov model" are used appropriately. Authors state that "sequences are simulated using a Markov chain". From the figure however, it seems that, a set of output sequence (i.e. s1, s2, s3, etc) are produced by applying the relevant transition matrix (F1 or F2, each with a 0.5 probability) to the seed sequence. Is this correct, or are the output sequences produced stepwise from one another (ie s3 is generated by "mutating" s2, which was generated by "mutating s1")? If all simulated sequences are derived directly from the seed, then the use of "Markov chain" is somewhat misleading (as only the first step of the chain is ever performed, though repeatedly). Similarly, can the authors explain why they refer to the model as a *hidden* Markov model? Specifically, the founder viruses and transition matrices, which would normally be the aspects of the model that would be "hidden", are instead treated as known. Given this, can the authors clarify their use of this terminology?

We agree that this was unclear and misleading. The reviewer is correct in that there is just one iteration from the seed per sequence and the transition matrix is specified. We no longer refer to this model as a Hidden Markov model. We sought to be more explicit in the revised text (as described above):

“The model was based on two training alignments, $F_{1}$ and $F_{2}$. $F_{1}$ corresponded to all the sequences descended from the major founder variant, i.e. the major founder lineage in a participant infected with multiple founder variants. $F_{2}$ grouped all sequences descended from minor founder variants in that participant (**Fig 1A**). Infections with multiple founder variants can be established with two founders that are clearly delineated; however, rare lineages and recombinant forms (based on the extant or unsampled founder variants) are often identified - these are sometimes found as singletons. While the model can be adapted to n founder lineages, for simplicity, here we considered the major founder lineage $F_{1}$ and grouped the other sequences in that participant (all closely phylogenetically related) as representing $F_{2}$. Hence, a group is not necessarily comprised of sequences descending from a unique founder variant but represents a genetically differentiable cluster with a divergent evolutionary pattern; identifying and distinguishing such clusters allows to recapitulate the diversity of acute infections with multiple founders without biasing towards one founder variant population. The percentage of non-consensus residues at each site ($\pi_{j}$) was calculated for $F_{1}$ and $F_{2}$ separately, giving a mutational landscape for each founder lineage (major and minor viral population) (**Fig 1B**). Next, a transition matrix, $\Theta$, was required to define the probability of each amino acid (or gap) transitioning into any other amino acid (or gap) (**Fig 1C**); the transition matrix is derived from a large set of independent circulating HIV-1 sequences (sampled from LANL HIV database). Finally, a single sequence, aligned to the training alignment reference frame, was required to seed the model (**Fig 1D**). The single seeding sequence corresponds to the design target and can be any sequence for which a set of multi-founder like antigens is to be derived, e.g. the consensus (or most common recent ancestor) from a set of sequences corresponding to a particular participant or to a specific HIV-1 subtype or Circulating Recombinant Form (CRF). At each iteration, $n$, site $j$ of the seeding sequence had a probability of mutating proportional to $\pi_{j}$ to a new residue defined by $\Theta_{j}$, where $\pi$ was randomly drawn from either $F_{1}$ or $F_{2}$ (**Fig 1E**). The model then outputs simulated sequences in an alignment, wherein equal numbers of sequences are derived from each founder lineage model (**Fig 1F**). Instead of considering the variability in a given participant, the model can also rely on a pooled dataset of major vs. minor founder lineages from multiple participants. In that case, at each site, the mutational pattern can be that of any individual in the pooled set (to retain within-host diversity characteristics and forbid inter-host transitions). The model was based on two training alignments, $F_{1}$ and $F_{2}$. $F_{1}$ corresponded to all the sequences descended from the major founder variant, i.e. the major founder lineage in a participant infected with multiple founder variants. $F_{2}$ grouped all sequences descended from minor founder variants in that participant (**Fig 1A**). Infections with multiple founder variants can be established with two founders that are clearly delineated; however, rare lineages and recombinant forms (based on the extant or unsampled founder variants) are often identified - these are sometimes found as singletons. While the model can be adapted to n founder lineages, for simplicity, here we considered the major founder lineage $F_{1}$ and grouped the other sequences in that participant (all closely phylogenetically related) as representing $F_{2}$. Hence, a group is not necessarily comprised of sequences descending from a unique founder variant but represents a genetically differentiable cluster with a divergent evolutionary pattern; identifying and distinguishing such clusters allows to recapitulate the diversity of acute infections with multiple founders without biasing towards one founder variant population. The percentage of non-consensus residues at each site ($\pi_{j}$) was calculated for $F_{1}$ and $F_{2}$ separately, giving a mutational landscape for each founder lineage (major and minor viral population) (**Fig 1B**). Next, a transition matrix, $\Theta$, was required to define the probability of each amino acid (or gap) transitioning into any other amino acid (or gap) (**Fig 1C**); the transition matrix is derived from a large set of independent circulating HIV-1 sequences (sampled from LANL HIV database). Finally, a single sequence, aligned to the training alignment reference frame, was required to seed the model (**Fig 1D**). The single seeding sequence corresponds to the design target and can be any sequence for which a set of multi-founder like antigens is to be derived, e.g. the consensus (or most common recent ancestor) from a set of sequences corresponding to a particular participant or to a specific HIV-1 subtype or Circulating Recombinant Form (CRF). At each iteration, $n$, site $j$ of the seeding sequence had a probability of mutating proportional to $\pi_{j}$ to a new residue defined by $\Theta_{j}$, where $\pi$ was randomly drawn from either $F_{1}$ or $F_{2}$ (**Fig 1E**). The model then outputs simulated sequences in an alignment, wherein equal numbers of sequences are derived from each founder lineage model (**Fig 1F**). Instead of considering the variability in a given participant, the model can also rely on a pooled dataset of major vs. minor founder lineages from multiple participants. In that case, at each site, the mutational pattern can be that of any individual in the pooled set (to retain within-host diversity characteristics and forbid inter-host transitions).”

e) The notation in the equations on p. 22 (step 3) are hard to follow, particularly the definitions of \Theta and \Theta'. If j is a site, k is an amino acid, and i_j is *also* a site, what does \Theta[i_j,] mean (the comma must be a typo), and what does \Theta'[i_{j=k}] mean? Then, what does \Theta[i_{j\noteq k}] mean? We think what you mean is that i_j is the amino acid at site j, though the rest of the notation is still unclear.

We thank the reviewer for pointing out these issues. We revised the method and clarified equations in the revised manuscript:

“A probabilistic model was developed to simulate Env sequences that replicated the variability of infections with multiple founder variants. The model is trained on two Env alignments, corresponding to the major (*F1*) and minor (*F2*) founder lineages identified in a given participant (the model can also be trained on a pooled set of individual lineages). A transition probability matrix, $\Theta$, is estimated by directly computing the transition rate between pairs of amino acids, including transition rates from/to gaps based on an alignment of empirical sequences. We suggest using a large dataset of independent sequences that would correspond to the subtype of the desired target vaccine candidate (i.e., subtype C sequences if the goal is a subtype C vaccine which implies that the seed sequence corresponds to subtype C). For each alignment,

1. The percentage of non-consensus residues was calculated for each alignment site.
2. For each site, $j$, percentage of non-consensus residues, $\pi_{j}$, an amino acid in the seeding sequence, $k$, and a transition probability, $\Theta_{k}$, the probability of transitioning from $k$ to $k’$ was written as

$$P\left( k'|k,\pi_{j},\Theta_{k} \right)=\frac{\Theta_{k}\left[ k \right]\pi_{j}}{\Sigma\left( \Theta_{k}\left[ -k \right] \right)}$$

where $\pi_{j}$ has an equal probability of being defined by $F_{1}$ and $F_{2}$. The new residue is then drawn from a multinomial probability distribution based on $P_{n}$. For each seeding sequence (e.g., a subtype C consensus sequence), whichever model is initially drawn to simulate the initial amino acid in a sequence is consistently applied to generate the following amino acids in that sequence. For the pooled sets, the less diverse major founder lineages were pooled together and the more diverse minor founder lineages constituted a second set. For each pooled founder lineage alignment, at each site j, $\pi_{j}$ was estimated as the maximum percentage of non-consensus residues in any individual alignment in that pool (i.e., this retained within-host diversity levels); however, this could be alternatively modeled such that, at each site j, $\pi_{j}$ was randomly drawn from an individual alignment in the pooled alignment.”

Moreover, in step 4, the authors' statement that a new amino acid was "drawn from a multinomial probability distribution" seems unnecessarily complex. In their setting, is N ever anything other than 1, and are n_1, n_2, ..., n_k ever anything other than a tuple of 0s and 1s where exactly one element is 1? It seems that it would be simpler to say that the new amino acid would be drawn according to the transition probability matrix.

The reviewer is correct that N=1 and we have revised the equation to reflect that, as well as rephrasing the explanation to say explicitly that the new amino acid is drawn from a multinominal probability distribution as defined by the transition probability matrix, which is only tuples of 0s and 1s if there is complete consensus at the site.

A clearer way to rewrite steps 3 and 4 may be to write \Theta_{k,l} for the transition probability of going from amino acid k to amino acid l (since j is used to denote a site). And, for each site j, make a transition with probability \pi_j, and stay put with probability 1-\pi_j. Then, if we have chosen to make a transition, we pick a new state according to the probabilities P(transition from i_j to k) = \Theta_{i_j, k}.

We are grateful for the reviewer for helping us rephrase this equation that is now in its revised form above and much clearer than before.

f) Speaking of \Theta and \Theta', is the term "transition rate matrix" used correctly here? As I understand it, this process is a discrete time process so these are "transition probability matrices", not "rate" matrices.

We thank the reviewer for this suggestion. We have used the term transition probability matrix in the revised manuscript.

g) Could the authors provide more detail on how the transition matrices are found. Are these directly computed from the empirical distribution of all mutations in each alignment, where the reference sequence is the consensus of the alignment (ie the founder virus)? If so, this should be stated directly.

We modified our strategy and are now using a matrix derived from a population level alignment. Here, the transition rates were computed directly on an alignment of 216 subtype C sequences sampled since 2011, which we have clarified in the revised manuscript:

Line 1671 (Methods):

“A transition probability matrix, $\Theta$, is estimated by directly computing the transition rate between pairs of amino acids, including transition rates from/to gaps based on an alignment of empirical sequences. We suggest using a large dataset of independent sequences that would correspond to the subtype of the desired target vaccine candidate (i.e., subtype C sequences if the goal is a subtype C vaccine which implies that the seed sequence corresponds to subtype C).”

4. How could this approach be used to develop a universal vaccine for HIV-1 group M? (lines 412-413)?

Technically, the approach can be used to develop a cocktail of minimally distant antigens centered around an HIV-1 group M sequence. While we think that obtaining an HIV-1 vaccine adapted to a single subtype is a better strategy and a necessary milestone before bridging to a group M vaccine, we note that other vaccine strategies include a group M insert and the goal for vaccine development is often to refer to elusive universal vaccines (for HIV-1 as for flu or coronaviruses). Our thinking is summarized in the revised discussion:

“While this model can also be used to design an HIV-1 group M vaccine cocktail, the idea of a successful universal HIV-1 vaccine is far-fetched when considering lessons from the past forty years of HIV-1 vaccine research.”

5. The discussion should feature a discussion of potential caveats/limitations of the approach.
We agree that this was an omission and have added a discussion of limitations to the revised manuscript:

“An important limitation of this method is its reliance on alignments from only six individuals to simulate multi-founder-like sequences. While each individual developed bnAbs, precise mechanisms behind the association between multi-founder variant diversity and the development of neutralization breadth are unknown. We tried to overcome this limitation by pooling founder lineage alignments to capture the scope of diversity found in acute infection in these broad neutralizers rather than relying on one individual to define a prototypical infection with multiple founder variants. Additionally, we separated sequences into two major and minor founder lineages - this does not account for the complexity seen in some infections with multiple founder variants which can include recombinants between extant and/or unsampled sequences and rare or even unique sequences. Hence, our model simplified the landscape of acute viral diversity in these individuals. Nonetheless, if datasets of increased depths are available, the model can be used to simulate the multitude of distinct sequences that can be identified in a set of hundreds of Env sequences from acute HIV-1 infection.”

Additional comments:

1) In figure 1E, the notation P(F1 U F2)= 0.5 is confusing. I assume this means that F1 and F2 have an equal (0.5) probability of being applied to a given output sequence. If so, this should be written as P(F1)=0.5; P(F2)=0.5

We have revised Figure 1E to make this clearer, as the reviewer suggested.

2) The results (lines 140-142) states that mutations occur... "such that at each iteration, , site of the seeding sequence had a probability of mutating proportional to to a new residue defined by , where and were randomly drawn from either 1 or 2 (Fig 1E).

But, this wording suggests that each *site* in a sequence could be subjected to either the mutation rules of F1 or the mutation rules of F2, independent of the other sites in that sequence. The legend of Figure 1E suggests the same. However, this approach could not produce the output shown in Figure 2. Instead, all sites within a given sequence must be subjected to either the F1 or F2 transition matrix... (?). Please clarify at what level (i.e. sequence or site) the choice of mutation rules is made.

The reviewer is correct. Each site is simulated based on either the F_1_ or F_2_ transition probability matrix, which is randomly drawn, but the transition matrix then remains constant for all subsequent sites, such that each site in a simulated sequence is drawn from either transition matrix but never both. We have clarified this in the revised manuscript:

“For a seeding sequence (e.g., a subtype C consensus sequence), whichever model is initially drawn to simulate the first amino acid in a sequence is consistently applied to generate the following amino acids in that sequence.”
