# Supplementary material for: Optimal sequence-based design for multi-antigen HIV-1 vaccines using minimally distant antigens
Source: PLoS Comput Biol. 2022 Oct 31;18(10):e1010624. doi: 10.1371/journal.pcbi.1010624 (PMC9621458; doi:10.1371/journal.pcbi.1010624)
Supplement: Attachment — Submitted filename: Response_PCOMPBIOL-D-21-02212R1_20220929.docx [file pcbi.1010624.s006.docx]

**Reviewer #1:** I thank the authors for the extensive work done to respond to the first round of reviews. My remaining comments are minor:

I think that the process by which a transition matrix is estimated from the Los Alamos data is either missing a citation or underdescribed (L150, L429)

We thank the reviewer for identifying this issue. We cited the corresponding reference:

Line 120-124. “Next, a transition probability matrix, $\Theta$, was computed according to the procedure described by Le & Gascuel (LG matrix) [39] to define the probability of each amino acid (or gap) transitioning into any other amino acid (or gap) (**Fig 1C**); the empirical transition probabilities were computed on an alignment of 172 subtype C Env sequences sampled since 2011 from the LANL HIV Database.”

Line 490-493. “A first-order Markov transition probability matrix, Θ, is estimated as described by Le and Gascuel (LG matrix) [39]; in brief, transition rates are directly computed between pairs of amino acids, including transition rates from/to gaps based on an alignment of empirical sequences.”

On line 164 the number of polymorphisms from training alignments is described as summarised by the mean, but in the legend of figure 2G the text indicates the median was used (L204).

The legend was correct. We changed the main text to match the legend for Figure 2G:

Line 187-190: “Similarly, the 95% confidence intervals of the median number of polymorphic sites per simulated sequence as well as the number of polymorphisms per sequence at CD4bs, V1-V2, V3 and MPER contact sites included the median number for training alignments for sequences simulated on each participant (Fig 2G).”

Also in figure 2G legend the "dashed lines" could refer just to the error bars (L204); this is a reference to the horizontal dashed lines presumably.

We changed the Figure 2G legend to refer to the “dashed whiskers” rather than “lines”: “Dashed whiskers indicate maximum values.”

I don't follow the argument that the bimodal or multimodal phylogenies in figure 4B must reflect recombination. These are simulated sequences and recombination was not explicitly modelled. If the argument is that recombination in the real alignment is the cause of these patterns, or that the simulation process generates patterns that mimic recombination, then this needs fleshing out.

We agree that the phylogenies in Figure 4B do not reflect recombination, as this was not modeled, and we corrected this sentence:

Line 273-274: “The sampled sequences produced phylogenies with bimodal or multimodal topologies (Fig 4B).”

The x-axis is missing from figure 4D.

We added a scale and label (“Env amino acid site”) to the x-axis in Figure 4D.

**Reviewer #2:** The manuscript by Lewitus et al is substantially improved and many of the concerns raised by reviewers have been addressed. There are still some points that would benefit from clarification, mostly related to the mathematical notation, and some additional minor suggestions to improve the MS, as follows

Related to the mathematical notation and model:

1. line 94-

- One improvement would be to use two separate names for \pi when it comes from F_1 and F_2. It appears that F_1(\pi_j) and F_2(\pi_j) are used to denote this (is this correct?), but it would be better to make this explicit, as this notation is introduced without explanation in lines 157-160

We appreciate the reviewer’s concern for clarity here. The reviewer is correct that F_1 (π_j )

and F_2 (π_j ) denote the percentage of non-consensus residues at site j for the F1 and F2 founder training alignments, respectively. We amended the text to make this clear:

Line 168-178: “One thousand sequences were simulated and trained on major and minor founder lineages alignments for each participant separately, where the percentage of non-consensus residues at each site, $\pi_{j}$, was defined by either the major founder lineages, $F_{1}\left( \pi_{j} \right)$, or minor founder lineages, $F_{2}\left( \pi_{j} \right)$.”

- The authors present the steps starting at the level of a single site, but this later requires some backtracking ("actually all of these sites follow the same template laid out for the entire sequence..." [paraphrasing]). Instead, a better starting point may be "at each iteration, choose one of F_1 and F_2." Everything flows better from there, since the entire sequence is be generated from that choice. (The same holds to some extent for lines 121-123.)

We amended this step in the description in line with the reviewer’s suggestion below:

Line 129-135: “At each iteration, n, sequence simulation was trained on either F_1 or F_2, such that site j of the seeding sequence had a probability of mutating proportional to π_j to a new residue defined by Θ_j, where π was drawn from either F_1 or F_2 (Fig 1E).“

line 110: Is the transition matrix is calculated for overall rate, or for each position? It appears that it is the former, but the notation at line 119 seems to imply that it is per-site, which is confusing.

The transition matrix is calculated for the overall rate (i.e., the rate at which one amino acid is likely to transition into any other amino acid or gap), as we describe in the revised text:

Line 120-124: “Next, a transition probability matrix, $\Theta$, was computed according to the procedure described by Le & Gascuel (LG matrix) [39] for the overall rate to define the probability of each amino acid (or gap) transitioning into any other amino acid (or gap) (**Fig 1C**); the empirical transition probabilities were computed on an alignment of 172 subtype C Env sequences sampled since 2011 from the LANL HIV Database.”

line 118: This is where the results could say: "At each iteration, choose one of F_1 or F_2. In this example, we will assume F_1 is chosen. Site j of the seeding sequence will have a probability of mutation proportional to F_1(\pi_j); if it mutates, it will mutate according to the transition matrix \theta".

We agree and, as described above, amended this step in the description:

Line 129-135: “At each iteration, n, sequence simulation was trained on either F_1 or F_2, such that site j of the seeding sequence had a probability of mutating proportional to π_j to a new residue defined by Θ_j, where π was drawn from either F_1 or F_2 (Fig 1E).“

line 119-120: Is there any reason to not simply do N/2 iterations with F_1 and then N/2 iterations with F_2? Doing so could also simplify this description.

While the approach taken in our simulations was to have equal numbers of sequences simulated under F1 and F2 (F1=N/2, F2=N/2), the method is flexible so that any ratio of F1 and F2 could be used and therefore we want to leave this option open to the user. We have amended the text to make this point:

Line 136-138: “Here we show equal numbers of sequences derived from each founder lineage model, however, the proportion of simulated sequences trained on each founder lineages alignment can be specified.”

Line 168-178: “One thousand sequences were simulated and trained in equal proportions on major and minor founder lineages alignments for each participant separately, where the percentage of non-consensus residues at each site, $\pi_{j}$, was defined by either the major founder lineages, $F_{1}\left( \pi_{j} \right)$, or minor founder lineages, $F_{2}\left( \pi_{j} \right)$.”

line 145: perhaps change to "across *the 6* participants" to make it clear that these summary stats are for the 6 participants from RV217 with the multiple founder infections (i.e. those in figure 2)

We amended this line:

161-163: “For the major founder lineage $F_{1}$, the mean percentage of non-consensus residues per site across the six participants was 0.06% (max=10%) and median pairwise amino acid diversity was 0.001 (min=0, max=0.005).”

lines 149-151: Suggestion to move this description of how \theta is defined to the first mention of \theta at line 110.

We have amended the first mention of theta so it is defined explicitly:

Line 120-124: “Next, a transition probability matrix, $\Theta$, was computed according to the procedure described by Le & Gascuel (LG matrix) [39] for the overall rate to define the probability of each amino acid (or gap) transitioning into any other amino acid (or gap) (**Fig 1C**); the empirical transition probabilities were computed on an alignment of 172 subtype C Env sequences sampled since 2011 from the LANL HIV Database.”

line 287: It is unclear how fig 5a-f show this. There is no scale on the x-axis.

We have added x-axis labels and scales for Figures 5A-F for clarity and amended the legend:

“In A-F, sequences are sorted along the x-axis by their y-axis value; the black line traces the value across sequences.”

line 439: Is the denominator needed? The normalization should presumably be inherent in \theta.

The denominator is needed so that the probability distribution sums to 1. For example, if at site j there is a 25% probability of transitioning from residue k to k’, then the probability of transitioning to k’ must be 1-P(k=k)=75%. Without the denominator, then P(k=k)+P(k!=k) will not sum to 1. We have amended the text to make this point:

Line 511-512: “The denominator, $\Sigma\left( \Theta_{k}\left[ -k \right] \right)$, is included to force the probability distribution to sum to 1, such that $P_{j}(k^{'}\neq k)$=1-$P_{j}(k^{'}=k)$.”

Also, is \theta_k[k'] "the probability of a transition from k to k', assuming such a transition happens"? If so, should this be \theta_k[k'] in the numerator?

The probability of transition from k into another amino acid is defined by P(k’), where this refers to the probability of transitioning into any other amino acid (or gap) and is therefore multinomial. For example, if the sum of transition rate probabilities from k to k’=80% (or 4% per new state) and pi_j=40%, then the probability of transition to each new state is 4%*40%/80%.

line 441: P_n has not been defined anywhere; this is just P, for this step. Perhaps you meant to say "At each step n" in line 434. This would also be a good place to say "first pick F_1 or F_2", or "we first simulate N/2 sequences based on F_1 and then N/2 sequences based on F_2".

Yes, this is correct and we amended the text to clarify:

Line 509-512: “where $P_{j}(k^{'})$ is calculated for each site *j*. For each simulated sequence, $\pi_{j}$ is trained on either $F_{1}$ or $F_{2}$ and at each site in the sequence, *j*, the new residue is then drawn from a multinomial probability distribution based on $P_{j}(k^{'})$.”

Other comments:

1. The manuscript is much more clear, but a brief accessible explanation of why "minimally distant" antigens are optimal for multivalent immunogen design, as early as possible in the introduction, would also be helpful

We added a clause in the introduction to state:

Line 82-87: “This led us to propose that a vaccine which would be constituted by multi-founder like, minimally distant antigens (differing by ~1% in Env) corresponding to the variability we observed in infections with multiple founder variants could initiate the induction of bnAbs. We hypothesize that a set of antigens that show minimal differences and differ primarily at surface sites, including sites that correspond to bnAb epitopes, would promote the maturation of neutralizing responses through toggle between variant epitopes.”

2. The authors' clarification in the response letter that the approach will primarily produce designs for immunogens used in *priming* vaccinations is helpful - as these are unlikely, on their own, to elicit NAb responses. However, I recommend that this is made more clear in the discussion (e.g. perhaps at the end of line 394 or concluding paragraph) as well as briefly in the abstract and/or author summary.

We revised the discussion. While at this stage of development of our vaccine candidate, we are most concerned with the initial priming immunization, this does not mean that the set of antigens could not be used for further immunizations. We want these decisions to be made based on experimental assays.

We revised the discussion as follows:

Line 450-454: “The fact that our vaccine design was derived from the variability seen in multi-founder acute infections suggests that this strategy with a cocktail of minimally distant antigens may be best suited as a priming immunization. Whether subsequent immunizations should consist of the same set of antigens or a subset of them, or one or more distinct antigens, will need to be evaluated with experimental assays.”

3. 5/6 of the training alignments are from individuals with CRF02_AE or A1, yet the model was used to produce simulated subtype C sequences (presumably as this the most prevalent subtype worldwide). Some readers may wonder whether this may be a concern; the authors may wish to briefly address this in the discussion.

We agree that this is an important consideration and have added this to the discussion:

Line 393-407: “While we chose to use as seeding sequence a subtype C consensus in order to design a subtype C vaccine candidate (because over half of the people living with HIV-1 live with subtype C), only one of the six infections we used in our pooled founder lineage alignment corresponded to subtype C (A1 (n=2) and CRF01_AE (n=3)). It has not been reported that patterns of variability in acute infections with multiple founders differ by subtype, nonetheless, it is possible that subtype C specific patterns exist and would not necessarily be captured by our approach.”

4. The colors in Figure 2 are re-used in a confusing way. e.g. - maroon is used for simulated data in 2C, D, but the legend of 2F indicates that maroon denotes training data. Similarly, blue is used in 2B, C and D to denote F2, but in 2F it denotes simulated data. MF is also the same blue color, but since this is from the pooled alignment, it may be better to use a different color entirely. It could be helpful to match Figure 3 as well.

We agree that this was poorly organized and changed the color scheme so that simulated data are shown in blue across figures, while training data are shown in pink. We also modified Figure 3 to use the same color code as for Figure 2.

5. It would be helpful to have slightly more information on the alignment of 172 subtype C sequences from LANL, as there appear to be far more than this since 2011. Was this restricted to single-genome amplified sequences? HIV RNA (or was DNA also allowed)? What is meant by "sequences were de-duplicated at 95% identity"?

All sequences sampled after and including 2011 that were available in the LANL database were downloaded and then curated through the following steps:

Line 481-484: “Sequences were excluded if the individuals had been vaccinated, if the sequence did not have a complete open reading frame, or did not have a sampling year. One sequence was downloaded per individual and sequences were removed if they were non-independent or an outlier. Hypermutated sequences identified with Hypermut 2.0 [73] (using https://github.com/philliplab/hypermutR) were removed with a Fisher’s exact test P<0.1. Sequences were de-duplicated at 95% identity.”

This resulted in 172 subtype C sequences. Sequences de-duplicated at 95% identity means that when two sequences were >95% identical, one of them was removed from the alignment.

6. recommendation to avoid using "infected" when describing people with HIV, instead use "person with HIV" or "person living with HIV" e.g. lines 42, 65, 368

We thank the reviewer for noting this. We corrected these occurrences.

7. small typos: line 42 comma should be a period. line 372: "breath" should be "breadth".

Thank you, we corrected these.
